# Advances in Photodynamic Therapy Based on Nanotechnology and Its Application in Skin Cancer

**DOI:** 10.3389/fonc.2022.836397

**Published:** 2022-03-16

**Authors:** Ping Zhang, Ting Han, Hui Xia, Lijie Dong, Liuqing Chen, Li Lei

**Affiliations:** ^1^ Department of Dermatology, Wuhan No. 1 Hospital, Tongji Medical College, Huazhong University of Science and Technology, Wuhan, China; ^2^ School of Materials Science and Engineering, Wuhan University of Technology, Wuhan, China; ^3^ Department of Hepatobiliary Surgery, Wuhan No. 1 Hospital, Tongji Medical College, Huazhong University of Science and Technology, Wuhan, China

**Keywords:** photodynamic therapy, photosensitizer, nanotechnology, nanoparticle, nonmelanoma skin cancer, metastatic melanoma

## Abstract

Comprehensive cancer treatments have been widely studied. Traditional treatment methods (e.g., radiotherapy, chemotherapy), despite ablating tumors, inevitably damage normal cells and cause serious complications. Photodynamic therapy (PDT), with its low rate of trauma, accurate targeting, synergism, repeatability, has displayed great advantages in the treatment of tumors. In recent years, nanotech-based PDT has provided a new modality for cancer treatment. Direct modification of PSs by nanotechnology or the delivery of PSs by nanocarriers can improve their targeting, specificity, and PDT efficacy for tumors. In this review, we strive to provide the reader with a comprehensive overview, on various aspects of the types, characteristics, and research progress of photosensitizers and nanomaterials used in PDT. And the application progress and relative limitations of nanotech-PDT in non-melanoma skin cancer and melanoma are also summarized.

## Introduction

Photodynamic therapy (PDT) is a non-invasive or minimally invasive treatment with verified safety, efficacy. In 2011, PDT has been listed as one of the primary methods for early esophageal cancer by NCCN in USA. Other tumors that have tried PDT strategy include gastric cancer, cholangiocarcinoma, squamous cell carcinoma on head and neck, early cervical cancer or precancerosis and urologic malignancies, and so on (1). At present, PDT is widely and maturely used in skin cancer and precancerous lesions with visual cosmetic effect.

PDT is based on photosensitizers (PSs), which intensely accumulate in pathological tissues, to produce cytotoxic substances under irradiation by light sources at a specific wavelength, and it acts on target tissues to kill pathological cells, induce microvascular injury, and stimulate immune responses. There are two types of photo-oxidative mechanism of PDT: PSs are converted from the singlet basic state into the excited singlet state after absorb the radiant energy, and then, partial energy direct PSs to the excited triplet state. On the one hand, PSs can transfer energy to the surrounding biomolecules, so reactive oxygen species (ROS) is further produced and tumor cells are destructed in this cascade of photodynamic reactions (Type I); on the other hand, PSs directly transfer energy to the ground state oxygen molecules, leading to the generation of singlet oxygen (^1^O_2_, Type II). Type II reaction is the most important process, as the amount of available oxygen decreases, Type I mechanism becomes significant (2). PDT has been mainly divided into tumor-targeted PDT, vascular-targeted PDT (VTP) and anti-microbial PDT. Most PDT are based on cellular-targeted photochemotherapy. VTP distinct from two other ways, and selectively disrupts vascular function by inducing oxidative damages to the vasculature, particularly endothelial cells, meanwhile, VTP-PSs remain predominantly confined within the circulation (3). Microorganisms thrive in well-organized biofilm ecosystems associated with infections. PDT overcomes biofilm producing pathogens by simultaneously acting on multiple target sites, such as damage to the cell membrane, DNA damage and protein/enzyme inactivation, and has been used against gram-negative and positive bacteria, fungi, viruses and parasites (4). In this paper, we reviewed the types, characteristics, and research progress of photosensitizers and nanomaterials used in PDT, and summarized the application outcomes of nanotech-PDT in non-melanoma skin cancer and melanoma.

## Research Status of PSs

Since the 1980s, PDT has been systematically studied, a series of PSs and light sources have been developed. PS is a compound that can absorb photons and transfer energy to molecules, thereby promoting chemical reactions without participating directly in them (5).

Commonly, PSs are classified into three generations. First-generation PSs (PSs1) mainly include porphyrin compounds which characterized with low chemical purity, poor tissue penetration and selectivity, and long residence time in body limit their utilization.

The structures of second-generation PSs (PSs2) are clear compared with those of PSs1, and the photosensitizing activity, absorption spectra, and penetration to deeply located tissues of PSs2 have been greatly improved (2, 6). PSs2 include derivatives of porphyrins (e.g., 5-Aminolevulinic acid, 5-ALA), chlorin (e.g., Temoporfrin/m-THPC, bacteriochlorin derivative) and metallic phthalocyanines (Pcs). 5-ALA is the precursor of protoporphyrin IX (PpIX, with strong photosensitive activity), thus 5-ALA has widely been applied to port-wine stain, severe acne, warts, cSCC, and precancerous lesions (7). Compared with porphyrins, chlorins have lower phototoxicity, large molar extinction coefficient, higher yield of ^1^O_2_ formation, and the maximum absorption wavelength at 650-800 nm, which make the depth of tissue penetration is up to 1 cm. Researchers synthesized the alkylether analogs of chlorophylla derivatives, 2-[1-Hexyloxyethyl]-2-devinyl pyropheophorbide-a (HPPH) characterized by single chemical structure, long excitation wavelength, good photostability. Chen et al. developed a rat PBPK model of HPPH, and demonstrated that the appropriate time window to conduct light exposure for the treatment of digestive cancer and skin cancer were 24-48 h and 48-96 h, respectively (8).

Pcs, which are large conjugated systems consisting of four pyrrole units linked by four N-atoms, can be functionalized with various materials, and the complexes formed by Pcs and non-transition metals (Zn, Al, Si) have photobiological activity against tumors. Some Pcs exhibit strong absorption at 750–900 nm, the light transmission ratio of melanoma is significant in this range, and the absorption increased nearly twice as much as that of the 630nm wavelength. As reported previously, Pcs demonstrated better efficacy in PDT against melanoma in terms of tumor cell photokilling and decreased dark toxicity than porphyrins because of their stronger reactive oxygen species (ROS) generation and better spectroscopic properties (9, 10). Pcs have strong hydrophobicity and a tendency to aggregate, thereby reducing the efficacy of PDT, but PSs2 can be re-functionalized by core or peripheral modification to overcome these drawbacks. Scientists synthesized sugar-bound zinc Pcs (ZnPcs) and found that the aggregation of C-glycoside–modified Pcs in solution could be greatly reduced, thus improving the stability of PSs.

**Figure 1:**
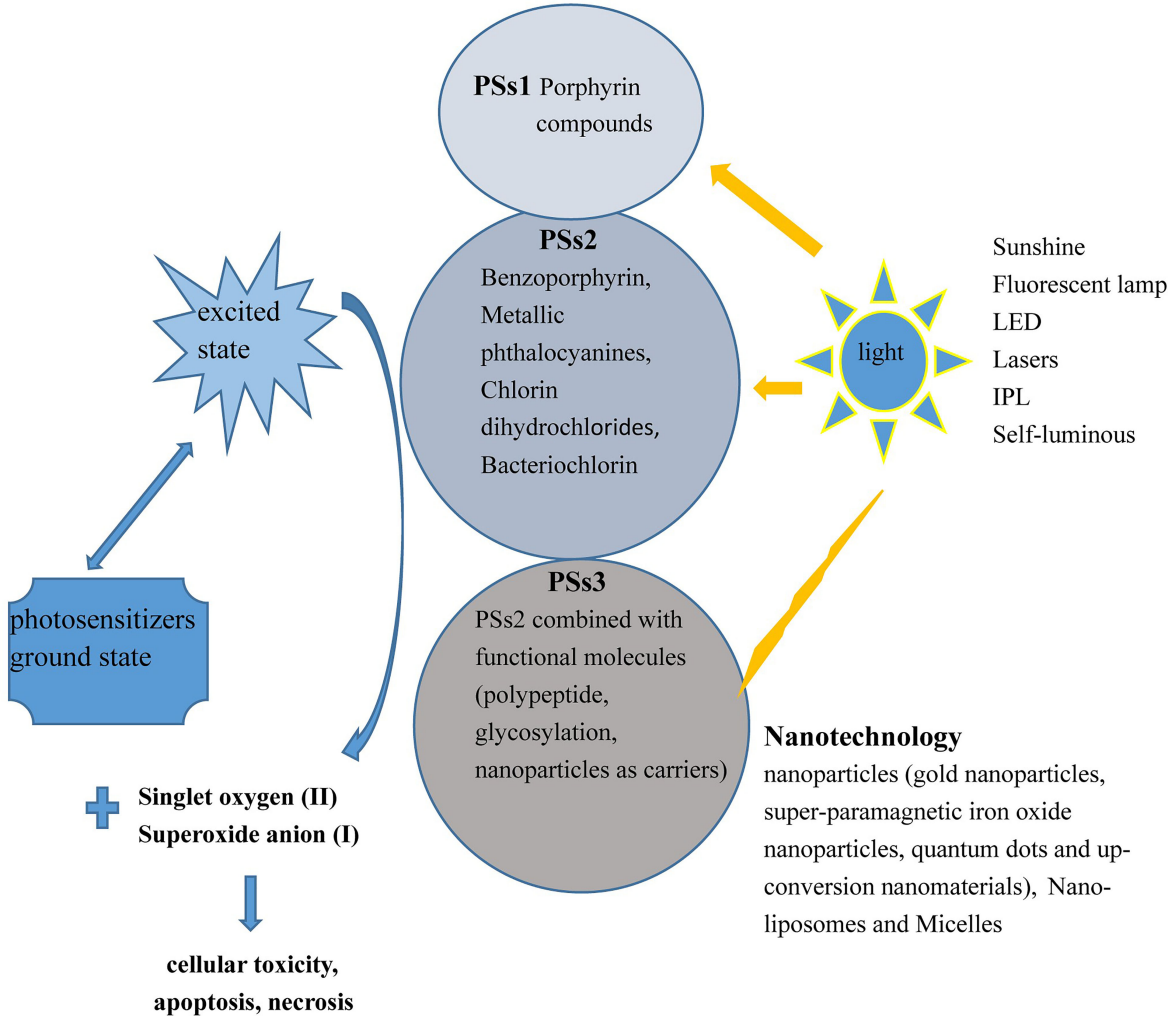
Action mechanism of PDT. First-, second-, and third-generation PSs absorb photons from different types of light sources and transfer energy (between the excited state and ground state) to generate 1O2 (II type) or superoxide anion (I type) to kill cells.

PSs3 are the products of PSs2 combined with functional molecules, including polypeptides, glycosylated compounds, and nanoparticles (NPs), as carriers to improve biocompatibility and targeting (11). The rapid proliferation of cancer cells requires a large number of carbohydrate compounds, and thus, glycosylated PSs inherently have enhanced targeting to cancer cells and better water solubility. Inactivating PSs are conjugated with a prodrug, such as a quenching agent or pH sensor, to activate the PSs to increase its specificity for cancer cells. Muttaqien et al. developed lower critical solution temperature polymers exerting pH-responsive phase transition for PDT. The PS-polymer conjugate exhibited significantly enhanced cellular uptake, and it augmented the efficacy of PDT in a tumor pH-selective manner (12, 13). Mechanism of action of PSs as presented in **Figure 1**.

## Nanotechnology

Polymerized PSs-NPs are the most widely studied PSs3, and they are usually produced *via* hydrophobic or electrostatic interactions between PSs and polymers, including polylactic acid, poly-D-lysine, polycaprolactone, and gelatin (14). The tumorous angiogenesis distinct from normal blood vessels in structure and morphology, with larger gaps between endothelial cells; and that, the lack of lymphatic in the tumors prevents lymphatic reflux, also known as enhanced permeability and retention (EPR) effect, consequently, NPs or other macromolecular substances can preferentially accumulate in tumor sites for a relative long time, thereby improving the efficiency of PDT and mitigating side effects in peripheral normal tissue.

PSs can be directly modified by nanotechnology or delivered by nanocarriers, such as NPs, nanoliposomes (LPs), and nanohydrogel particles. NPs include gold NPs (GNPs), super-paramagnetic iron oxide NPs (SPIONs), quantum dots (Qds), and upconverting nanomaterial, and so on (15).

### NPs

NPs can be combined with PSs to obtain novel nano-PSs. Compared with conventional PSs, nano-PSs have many advantages, such as a small size, large specific surface area, high catalytic efficiency, a higher number of active centers, and strong adsorption capacity (16).

GNPs with good biocompatibility and hypotoxicity are generally used as passivating agents for SPIONs and carriers for PSs or anticarcinogen (17). GNPs modified by polyethylene glycol (PEG) have improved solubility and the ability to resist protein adsorption and avoid removal by the reticuloendothelial system, thereby prolonging the residence time of drugs *in vivo*. GNPs are divided into passive and active types according to whether they play a tumor-photosensitizing role in PDT. Passive GNPs, which are mainly used for drug transportation, have no substantial effects on the efficiency of PSs. Conversely, active GNPs can effectively absorb light energy to increase PS excitation and ROS production. The representative active GNPs are plasma active NPs, such as gold nanorods (AuNRs), shells, and cubes (18). Some GNPs have strong plasmonic resonance bands, which can effectively absorb near-infrared light and convert photons into heat energy, thus causing irreversible high-temperature ablation of cancer cells (19).

GNPs have been explored in various biological applications because of their chemical inertness and minimal toxicity. Li et al. (20) reported hexadecyl dimethylammonium bromide-coated AuNRs with positive charges that were bound to sulfonated aluminum Pcs (AlPcS) with negative charges to form AuNRs-AlPcS *via* electrostatic force. These constructs had 5-fold stronger fluorescence, and they caused typical light dose-dependent PDT-mediated damage in QGY liver cancer cells. This synergistic reaction simultaneously improved photodynamic detection and treatment efficacy.

Qds are powerful and versatile biological imaging probes. Qds possess wide light absorption bands, two-photon absorption cross-sections, and light resistance. The chromophores in biological system are quite complex. Photons with different energy densities may resonate with different chromophores to produce different electron transitions and photoluminescence (PL) reaction, so the tunable PL spectrum of Qds can match the requirements for diverse tumor tissues (21). However, Qds have lower ROS yields than traditional PSs, and some contain toxic cadmium, therefore, Qds and PSs are complementary modalities, and their advantages are magnified when they are used in combination. Samia et al. (22) proposed the concept of “Qds-PS for PDT”. Specifically, Qds as energy donors produce fluorescence resonance energy transfer (FRET) with traditional PSs as acceptors. The non-covalently linked Qds-Pc4 complexes were found to increase ROS production.

To date, a series of Qds-PS systems have been developed, such as Qds-porphyrins, Qds-Pcs, and Qds-organic or inorganic dyes. Qds with high quantum yield generally have poor water solubility, and thus, surface modification is required to improve their solubility. Karabanovas et al. (23) prepared CdSe/ZnS Qds loaded with chlorin e6 (Ce6) and coated with phospholipids, and the water solubility and colloidal stability of these compound were greatly improved.

PSs conjugated to water-soluble Qds result in FRET from Qds to PSs, thus improving the effect of PDT (24). Yueshu et al. (25)coordinated CuInS2/ZnS(CIS/ZnS) Qds with 5-ALA to form CIS/ZnS–5-ALA conjugates *via* chemical bonding. Their analysis revealed that the FRET efficiency of the conjugates was 58.49%, and the viability of cells *in vitro* was less than 40% after 800- and 1300-nm femtosecond excitation. Li et al. (26) reported the conjugation of AlPcSs with amine-dihydrolipoic acid-coated Qds *via* electrostatic binding, and compared with free AlPcSs, the AlPcS-Qd compounds had a greatly increased uptake rate in human nasopharyngeal carcinoma cells (KB cells) and achieved FRET with 84% efficiency.

SPIONs are magnetic particles with iron oxide NPs (mainly including Fe_3_O_4_ and Fe_2_O_3_) as their cores. The sizes of SPIONs have a linear relationship with their magnetization intensity indices (27). Studies illustrated that SPIONs with a crystal nucleus diameter < 10 nm exhibit superparamagnetism. However, the minimum pore size of microvessels in normal tissues is approximately 10 nm, and thus, an excessively small particle size may lead to drug leakage and affect the sedimentation rate. Oppositely, >200-nm particles in tissues were easily engulfed by the phagocytic system (28), therefore, an appropriate size is needed in the application of SPIONs. Magnetic NPs of 10–100 nm in size, which have the best stability and magnetism, can act as effective drug carriers (29). They have been successfully used for delivering porphyrins and non-porphyrin precursor drugs to targets during PDT, thereby promoting the development of multi-function SPION-PS conjugates (30).

To obtain better properties, SPIONs are often surface-modified and then further functionalized with a variety of functional groups (31), which mainly include synthetic polymers (e.g., PEG), neutral polymers (e.g., dextran, chitosan), inorganic metals, inorganic oxides (e.g., silica), and bioactive molecules (e.g., liposomes). Leyong et al. (32) developed multi-component nanocomplexes composed of SPIONs, upconverting NPs (UCNPs), and AlPcS4, which could kill 70% of MCF-7 cells under irradiation by a 980-nm laser. He et al. (33)synthesized Fe_3_O_4_@GNPs bound to hemagglutinin as bimodal contrast agents for imaging in colorectal cancer models. Wei et al. (34) constructed an integrin receptor-targeted cRGDfK-SPION nanomolecular probe *via* chemical methods to image HepG2 cells with strong αVβ3 integrin receptor expression, and the results indicated that cRGDfK-SPION significantly accumulated in HepG2 cells *in vitro*.

Silica-based mesoporous materials, which benefit from their special structure-ordered pore network, can induce promising controlled loading and release of PSs or drugs. Multifunctional nanocomposites conjugated with Pc4-magnetic NPs have been used as potential drugs for PDT application. Gauta and colleagues reported (35) ZnPc linked to folic acid and amine-functionalized magnetic NPs to form ZnPc-AMNPs-FA conjugates, and these constructs both localized at tumor sites with the aid of an external magnetic field and possessed the advantages of targeted PDT modes combined with MRI. Refilwe et al. (36) developed ZnTPPcQ-DNDs-BODIPY (conjugated with ZnPc, nanodiamond, and halogenated BODIPY) as PSs with higher ^1^O_2_ quantum yield in water solution by testing fluorescence lifetimes from time-correlated single photon counting methods, thus, the three-component nanoassembly worked better than the free individual components.

UCNPs can convert the near-infrared light (NIR) with strong tissue penetration into UV or visible light (VIS), providing a light converter for PDT activated by NIR, which is expected to solve the problem of shallow tissue penetration depth in traditional PDT. As a unique light conversion material, UCNPs have the strengths of low toxicity, a narrow emission band, long emission life, photobleaching resistance, non-autofluorescence background, and display great potential in biological imaging, biosensing, and drugs release control. In addition, UCNPs as carrier-loaded PSs can avoid the easy agglomeration of hydrophobic PSs. UCNPs can be effectively enriched in tumor sites by the EPR effect (37, 38).

SiO_2_ coating, self-assembly and covalent bonding are three common methods to construct UCNP-PDT system. Zhang et al. (39)constructed mesoporous silica-coated UCNPs that could convert deeply penetrating near-infrared to visible wavelengths. In this study, the composites loaded with two types of PSs (MC 540 and ZnPc) were designed to improve the ROS yield and enhance the PDT effects by using the polychromatic emission of UCNPs excited with 980-nm light source. Chen et al. (40)constructed complexes (UCNP@BSA-RB&IR825) coated with bovine serum albumin (BSA), which improved the solubility and stability of these complexes in the physiological environment. Emphatically, rose bengal (RB) and IR825 were simultaneously loaded in the BSA layer. Under NIR, these dual dye-loaded UCNPs were used for binary model imaging *in vivo*; meanwhile, photothermal therapy (PTT)/PDT synergistically ablated cancer cells. In UCNP-PDT system, UCNPs and PSs act as energy donor and acceptor, respectively. Ce6 and UCNP-NaYF4:Yb,Er@CaF2 were assembled into tumor pH-responsive composites, and after 980-nm excitation, NaYF4:Yb,Er@CaF2 produced upconversion fluorescence at 675 nm, which prompted Ce6 to produce ROS to selectively kill deep tumor tissues in a lower pH microenvironment (41). The proximity load and high content of PSs on UCNPs is the key to improve the energy transfer efficiency of UCNP-PDT.

### LPs and Micelles

LPs are single or multi-lamellar nanosystems spontaneously formed by phospholipids dispersed in aqueous medium, and LPs accumulate passively in tumor cells *via* the EPR effect (42). Some LPs are biocompatible and biodegradable, and they can bind with a variety of hydrophilic and hydrophobic drugs. Among these moieties, PEGylated LPs exhibit longer blood circulation, and therefore, they have been widely investigated for drug delivery application (43).

Liposomal preparation can effectively prevent the aggregation of PSs, thereby enhancing their photoactivity. ICG green-loaded PSs that released the dye in response to NIR displayed greater ability to suppress tumor growth in a breast cancer model than the free dye (44). Scientists had prepared ICG-coated photothermal-sensitive LPs that damaged DNA *via* PDT-mediated ROS production and exerted strong inhibitory effect on triple-negative breast cancer cell lines (MDA-MB-468, HCC-1806) (45). The coupling of targeting moieties to the surface of LPs enabled LPs to selectively bind to specific tissues or cell types.

The efficiency of chemotherapy is restricted by the limited uptake of anti-cancer drugs in a bioavailable form. LPs can integrate PSs and drugs simultaneously to create co-delivery systems. It has been reported that doxorubicin (Dox)-loaded porphyrin-phospholipid LPs (Pop-LPs-Dox) had long circulation half-life and storage stability. Pop-LPs-Dox were injected into mouse subcutaneous pancreatic xenografts (3–7 mg/kg Dox) under near-infrared irradiation, and the results revealed 7-fold greater Dox accumulation in tumors (46). Because of rapid tumor growth and increased oxygen consumption, hypoxia microenvironment enormously restrict the efficiency of PDT. Shi et al. (47) encapsulated catalase, lyso-targeted NIR-PS, and Dox in LPs to form complexes that enhanced oxygenation in tumors and promoted ^1^O_2_ production through the catalysis of intratumoral H_2_O_2_, thus significantly inducing apoptosis in tumor cells.

Micelles are self-assembled nanoscale colloidal particles with a hydrophobic core and hydrophilic shell. PS-loaded polymeric micelles are characterized by a long bleeding cycle time and high accumulation in diseased tissues. Many PSs can be used in combination with LPs or micelles for PDT (48). Wu et al. (49)confirmed that porphyrin- and galactosyl-conjugated polymer micelles had better targeting and PDT efficiency in HepG2 cells. Elbayoumi et al. (50) investigated that tetraphenylporphyrin (TPP)-loaded PEG-PE micelles had greater cytotoxicity than TPP alone and better targeting in MCF-7 cells. Deng et al. (51)constructed PEGylated iridium-based nanomicelles (IP600−IP4000 NPs) and found that the micellar stability increased with increasing PEG length and cellular uptake decreased with increasing PEG length. IP2000 and IP4000 NPs had excellent biocompatibility, and further analysis revealed that IP2000 NPs displayed higher therapeutic efficacy in PDT.

Micelles carrying PSs may cause phototoxicity during blood circulation, such as damage to endothelial cells and adjacent vascular cells; therefore, it is necessary to diminish side effects in the untargeted region (52). Li et al. (53) designed PEG-b-poly (caprolactone) micelles containing pheophorbide A (PhA) as the PS and the antioxidant β-carotene (CAR) *via* simple physical incorporation. CAR removed ^1^O_2_ produced by PhA in blood circulation and reduced the phototoxicity. When the micelles were ingested by tumor cells, the spatial isolation of PhA and CAR hindered the scavenging of ^1^O_2_, resulting in remarkable tumor cell death. This strategy ameliorated the accuracy and safety of PDT in cancer treatment.

Regarding the advantages and drawbacks of nanomaterials, GNPs are easy to prepare, but their removal is slow for their poor biocompatibility. Qds can permit combined labeling, detection, and treatment, but some Qds contain toxic heavy metals. UCNPs are limited to *in vitro* use because of their weak drug loading and low upconversion efficiency. Micelles are commonly used to deliver hydrophobic drugs, but their stability *in vivo* needs to be improved.

## Application of Nano-PDT in Skin Cancer

On the basis of pathological findings, squamous cell carcinoma (SCC), basal cell carcinoma (BCC), and malignant melanoma (MM) are among the most common skin cancers. The rest of skin cancer, such as Paget’s disease, sarcoma, and cutaneous T/B-cell lymphoma, are relatively rare. According to the origin of cells, skin cancer can also be divided into two types: MM and non-melanoma skin cancer (NMSC). Epidemiological analyses revealed an estimated 300 skin cancer cases per 100,000 people worldwide in 2018 (54). Skin cancer is common in Caucasians because of environmental pollution and other adverse factors, but the incidence of skin cancer in China is also increasing annually. Cutaneous SCC (cSCC) accounts for approximately 20% of skin tumors (55). Although primary cSCC can be successfully treated with surgery, whereas, 5% of patients with cSCC develop metastasis or local recurrence after complete resection.

The incidence of MM is increasing rapidly. Each year, there are an estimated 280,000 new cases of MM and more than 60,000 deaths worldwide. Globally, there are significant differences in the morbidity and mortality of MM, and these differences are mainly related to the timing of its detection and treatment (56). Traditional treatments for MM include surgery, chemotherapy, and radiotherapy. In recent years, targeted therapy and immunotherapy have made significant progress in extending survival and improving the quality of life of patients. Topical therapy can enhance the local drug concentration in diseased regions, meanwhile it has less potential toxicity than systemic drug therapy, making it an effective supplement to systemic therapy.

In 2010, Norwegian Photodynamic Therapy Group and medical specialists presented guidelines for the practical use of topical PDT in NMSC (57). PSs can preferentially aggregate in lesions and produce specific biological effects. This important property gives PDT great promise for the targeted therapy in skin cancer. PSs are delivered to patients topically, orally or intravenously.

5-ALA and methyl aminolevulinate (MAL, increased lipid solubility compared with ALA) have been approved by the FDA for dermatologic indications, but as prodrugs, they must be converted into active PpIX, which preferentially localize in endoplasmic reticulum to induce cell death upon irradiation. The major absorption peak of PpIX occurs in the blue light range at 410-420 nm, but other wavelengths with smaller absorption peaks can also be used to activate 5-ALA. A blue light source, potassium titanyl phosphate laser, pulsed dye laser, and intense pulsed light have broad applications for PDT. Red light with a wavelength of 630 nm (e.g., helium-neon laser) also served as a light source in some investigations (58). Generally, the depth of light penetration into skin increases at longer wavelengths, and thus, red light and NIR are more effective for thicker lesions.

The optimization of PDT depends on appropriate light parameters, oxygen, the kinetic distribution and concentration of PSs in tissues, but the low skin penetration of 5-ALA limits the efficacy of PDT. Champeau et al. (59) reviewed chemical (containing nanotechnology) and physical strategies for improving PSs/drugs penetration and PpIX metabolism in skin cancer.

### Skin Barriers and Drug Penetration

Methods for enhancing topical drug penetration include changing the thickness of the stratum corneum and chemical absorption of skin barriers, and among these strategies, ablative fractional laser (AFL) can create microchannels in the skin surface to facilitate PS uptake. Scientists used three groups of far-infrared wavelength lasers (CO_2_ laser with 10,600 nm wavelength, Er : YAG laser with 2940 nm, and Er : YSSG laser with 2790 nm) to create vertical micropores in the epidermis to increase drug delivery *via* AFL; meanwhile, the depth of the micropores could be controlled by the laser energy density. Anderson’s group conducted a randomized clinical trial (60) using AFXL (10 mJ per pulse, 0.12 mm spot, 5% density; UltraPulse-DeepFx, Lumenis Inc.) combined with MAL cream to treat 212 patients with actinic keratosis (AK, severity grade I–III). After 3 months of follow-up, AFXL-PDT was significantly more effective than PDT for all AK grades. According to the theory of fractioinal photothermolysis, AFL equipped with a picture generator, which disperse the concentrated spots into dozens to hundreds of smaller focal spots, can vaporize the target tissues in matrix mode, so that the skin tissues between heat damage areas are not affected, simultaneously promote skin healing. Although AFL improved the permeability of PSs, they have some drawbacks, such as secondary skin infection, inflammatory hyper/hypopigmentation, and scarring (61).

Microneedles (MNs) with different materials and shapes can improve cutaneous delivery. Moreover, MNs shorter than 900 μm in length do not damage deep blood vessels and nerves, leading to low risks and minimal pain (62). Wu and co-workers constructed an analgesic MN patch that features dissolvable MNs to transdermally deliver calcitonin gene-related peptide antagonist to treat localized neuropathic pain. The study demonstrated that AMN patches did not cause skin irritation or systemic side effects (63). A randomized clinical trial confirmed that expedited MN-assisted PDT for the *in situ* treatment of AK significantly shortened the incubation time to 20 min achieved 76% clearance, similar to the findings for conventional 1-h 5-ALA incubation (64).

In addition to AFL and MNs, microdermabrasion, curettage, and keratolytics as physical methods can also promote the permeation of PSs/drugs into skin by reducing the thickness of the stratum corneum.

Extensive studies have been conducted in the past 20 years to explore the superiority of 5-ALA PDT and MAL PDT with different light sources in the *in situ* treatment of AK and cSCC compared with other topical therapy modalities, such as 5-FU, imiquimod, and cryotherapy (65). PDT is recommended to treat superficial and thin nodular BCC (thickness < 2mm), and the combination of superficial debridement can appropriately extend the depth of PDT (66). Roozeboom et al. (67) compared the long-term effectiveness of fractionated 20% 5-ALA PDT with prior partial debulking versus surgical excision in nBCC. The result revealed that the recurrence rate was higher in the 5-ALA PDT group than in the surgery group.

MAL exhibits lipotropic effects as well as better selectivity and deeper skin penetration than 5-ALA. Clinical trials (68)demonstrated the efficacy of MAL PDT (red light is preferred as the excitation light) in the treatment of severe acne, non-hyperkeratotic AK on the face and scalp, Bowen’s disease, and NMSCs such as BCC that are not suitable for conventional surgery. Rhodes et al. (69) confirmed that MAL PDT is not inferior to surgery for nBCC after 3 and 24 months of follow-up. Szeimies et al. (70) reached the same conclusion after 3 months of follow-up, but the recurrence rate was significantly higher in the MAL PDT group than in the surgery group at 12 months.

### Prodrugs With Enhancing Strategies

PpIX has strong fluorescence and photosensitizing activity, and some tumors exhibit elevated 5-ALA–mediated PpIX levels; thus, increasing PpIX levels can improve the effects of PDT against tumors. Xue et al. (71) summarized the strategies for enhancing 5-ALA–based tumor detection and PDT. Iron chelators can remove ferrous iron and prevent the conversion of PpIX into heme, thus increasing PpIX accumulation.

**Figure 2:**
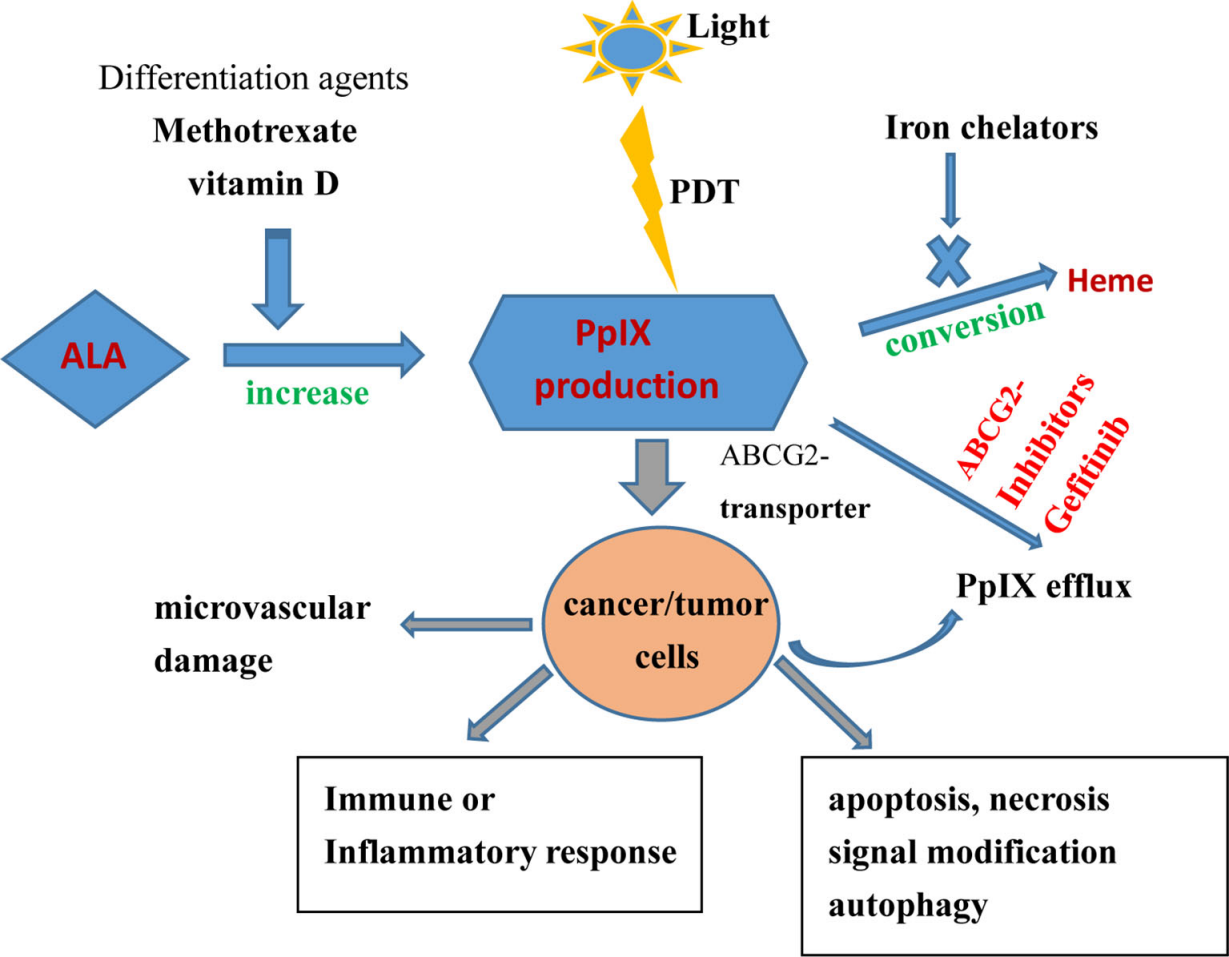
Effects of topical PDT on cancer/tumor cells and therapeutic strategies for enhancing ALA-based tumor therapy (increasing PpIX synthesis, reducing PpIX conversion, and inhibiting PpIX efflux).

Some differentiation agents, such as methotrexate and vitamin D, can increase 5-ALA–mediated PpIX production and improve 5-ALA PDT outcomes by upregulating CPOX. Anand et al. (72) found that using oral vitamin D3 prior to PDT, which increased PpIX levels by 3–4-fold, enhanced PDT-mediated cell death by 20-fold in cSCC without inducing unfavorable phototoxicity in normal tissues. PpIX is an endogenous substrate of the ABCG2 transporter. Sun et al. (73) confirmed that gefitinib (a potent ABCG2 inhibitor) could inhibit ABCG2-mediated PpIX efflux from malignant brain tumor cells, thereby increasing intracellular PpIX levels and the effect of PDT, as presented in **Figure 2**.

Collier and Rhodes (74) provided an outline of knowledge based on an analysis of randomized controlled trials of topical PDT in BCC, introduced the mechanism of action of topical PDT, described the use 5-ALA/MAL as prodrugs, and discussed the development of strategies for enhancing penetration and optimizing PpIX accumulation (e.g., epigallocatechin-3-gallate, iron chelators). The correlation between BCC subtypes and the effect of PDT (superficial low-risk BCC displayed higher complete response rates to topical PDT than other subtypes of BCC) was revealed, and a comparison of topical PDT with other topical methods (e.g., surgical excision, cryosurgery, topical 5-FU, imiquimod) was reported.

### Nanotechnology in PDT for NMSC Treatment

Due to the limited skin penetration and inferior luminescence efficiency of 5-ALA/MAL, microvehicles and nanodrug delivery systems such as NPs, LPs, and micelles have been developed (**Table 1**).

**Table 1:** Studies about nano-PDT for NMSC treatment.

References, Years	Characteristics of PSs	Wavelength of excitation	Time-window between PSs application and irradiation	Efficiency	Adverse effects	Design
Buchholz J, et al. 2007 (76)	LP formulation of meta-(Tetrahydroxyphenyl)Chlorin (m-THPC)	652 nm diode laser	0.15 mg m-THPC/kg body weigh, cephalic or femoral vein injection, treatment time (200s)	1-year control rate was 75%	local erythema and edema in 15% of the cases	cats-cSCC, in *vivo*
Flickinger I, et al. 2018 (77)	LP phosphorylated m-THPC, dosage of 0.15mg/kg body weight	652 nm diode laser	6 hours after injection, 10J/cm^2^ treatment time of 100 s, 20J/cm^2^ within 200s	overall response rate was 84% with a mean progression-free interval of 35 months	Local erythema and edema in 42% of cats shortly after PDT	cats-SCC, in *vivo*
Wozniak M, et al. 2021 (78)	curcumin encapsulated in hydrogenated soy phosphatidylcholine LPs	blue light 2.5 J/cm^2^	4h of pre-incubation	10 μM concentration caused decreased viability in SCC-25 (34%), MUG-Mel2 (27%) and HaCaT (11%)	/	melanoma MUG-Mel2, SCC-25, HaCaT cells, in *vitro*
Master A, et al. 2013 (79)	silicon phthalocyanine-4 (Pc 4) packaging within polymeric micelles that are surface-decorated with GE11-peptides	1) photoirradiated 400s 2) 672 nm two-beam split diode laser	1) incubation times varying from 2h to 24h 2) 2days after 0.01 mL/g bolus tail vein injection	1) enhanced Pc 4 uptake and significant cell-killing 2) enhanced Pc 4 uptake and regressed tumors	/	1) EGFR-overexpressing SCC-15, and in *vitro*. 2) human tongue carcinoma xenograft tumor mice model in *vivo*
Wang X, et al. 2015 (81)	ALA-loaded polylactic-co-glycolic acid (PLGA) NPs	helium–neon laser (632.8nm)	topically cream containing 0.8% ALA, combination of microneedles	enhanced protoporphyrin IX production; decreased tumor sizes	/	UV-induced mice cSCC
Keyal U, et al. 2018 (82)	ZnPc-loaded chitosan/mPEG-PLA NPs	670 nm diode laser	after microneedles-pretreatment, topical application	the effect of PDT was significantly better with Z-CPP compared to free ZnPc	no dark toxicity, without systemic toxicity	primary cultured cSCC cells from UV-induced SKH-1 SCC mice, in *vitro* and in *vivo*
Morton CA, et al. 2018 (83)	BF-200 ALA gel vs. MAL cream	red light (635nm)	topical application, two PDT sessions 1 week apart	complete responders: 93·4% in ALA droup, 91·8% in the MAL group		randomized, multinational, noninferiority, phase III trial in Germany and the U.K non-aggressive BCCs
Salmivuori M, et al. 2020 (84)	Hexyl aminolevulinate (HAL2%) vs. MAL 16%, BF-200 ALA 7.8%		topital application after 3h, illuminated 7min and 24s	lesion clearance: 93.8% for MAL, 90.9% for BF-200 ALA and 87.9% for HAL	pain, swelling, oedema, erythema and haematoma in the treatment area	prospective, non-aggressive BCCs

Studies reported that LPs increase the penetration of 5-ALA into skin relative to free 5-ALA and limited drug absorption into systemic circulation, thus reducing cytotoxicity and phototoxicity after treatment (75). Researchers assessed the possible toxicities of a liposomal formulation of PS (m-THPC) in feline cSCC. All cats responded to therapy (0.15 mg m-THPC/kg injection into the cephalic or femoral vein), and overall 1-year control rate was 75%. This outcome illustrated that liposomal PS was safety and efficacy (76). Scientists (77) investigated the long-term outcomes and prognostic factors of feline cSCC following the intravenous injection of liposomal phosphorylated mTHPC under irradiation with a 652-nm diode laser, and the overall response rate was 84% (complete remission, 61%; partial remission, 22%) with a mean progression-free interval of 35 months. Woźniak et al. (78) analyzed the photosensitizing effect of curcumin-loaded LPs acting in MUG-Mel2 (melanoma), SCC-25 (SCC), and HaCaT (normal keratinocytes) cells *in vitro*, revealing that the liposomal formulation improved the photosensitizing properties of curcumin-mediated PDT in skin cancers and reduced toxicity in normal keratinocytes. Alyssa et al. (79) reported that a nanoformulation of EGFR-targeted Pc4 packaged with polymeric micelles underwent faster and more extensive uptake in EGFR-overexpressing H&N SCC-15 cells, subsequently, they tested the PDT response of the Pc4 nanoformulation in subcutaneous SCC-15 xenografts in mice, and found that the higher level of Pc4 intra-tumoral uptake corresponded to the cell-study, thus improving PDT efficacy *in vitro* and *in vivo*.

PDT is limited by the sample size and tumor depth. For example, cSCC limited to the papillary dermis can be treated by PDT. To overcome these hurdles, pretreatment with AFLs or MNs and the encapsulation of PSs by LPs or other nanoplatforms have been attempted with remarkable efficacy (80). NPs have also been used as PDT enhancers because nanocarriers can passively accumulate at tumor sites *via* the EPR effect. Wang et al. (81)prepared 5-ALA–loaded polylactic-co-glycolic acid (PLGA) NPs as a cream formulation for application onto tumor surfaces in combination with helium–neon laser irradiation, finding that 5-ALA–PLGA NPs mediated PDT more effectively than free 5-ALA at the same concentration in cSCC. Loaded ZnPcs on chitosan/methoxy polyethylene glycol-polylactic acid (CPP) was used to form NP-(Z-CPP), and the dark toxicity and efficacy of topical PDT in SCC was examined using *in vivo* and *in vitro* tests. The results demonstrated that the compounds exhibited no dark toxicity, and the efficacy of PDT was significantly better using Z-CPP than using free ZnPcs (82).

PDT is an established treatment for low-risk BCC, mainly including primary nodular and superficial BCC. A randomized phase III trial evaluated the non-inferiority of BF-200 5-ALA (nanoemulsion gel containing 5-ALA) to MAL (cream containing MAL) in the treatment of sBCC or nBCC *via* PDT. The complete response rate in the BF-200 ALA group was 93.4%, compared with 91.8% in the MAL group, and BF-200 ALA-PDT proved non-inferior to MAL-PDT (83). Salmivuori et al. (84)conducted a prospective and double-blinded trial and confirmed that low-concentration hexyl aminolevulinate and BF-200 5-ALA had similar efficacy and tolerability as MAL in PDT for non-aggressive BCC.

### PDT Based on Nanotechnology for Melanoma

Melanoma is an invasive and aggressive skin cancer, and can be classified in pigmented and unpigmented-type. In 2019, The European Interdisciplinary Guideline on melanoma made the recommendations on cutaneous melanoma diagnosis and treatment, and confirmed the primary treatment of melanoma is surgical excision, PDT hasn’t been mentioned in adjuvant therapy (85). However, PDT has become an effective treatment for superficial and localized cutaneous MM in some published literatures.

Tumor cells can induce the formation of their own defense system to some extent, and form an antioxidant defense system under the oxidative stress triggered by PDT. Nano-PSs have the advantages of high ROS yield, easy modification and good stability, and can overcome PDT resistance. Tang et al. observed that NPs could kill tumor cells directly *via* oxidative stress, DNA damage, and cell membrane damage in MM and demonstrated that they bind chemotherapeutic drugs or nucleotide fragments as carriers *via* electrostatic force or hydrophilic/hydrophobic interactions to enhance the biocompatibility and targeting of nanodrugs (86). Small interfering RNA (siRNA) can block gene expression to achieve high gene silencing for tumor treatment, but siRNAs are not stable, permitting their easy degradation in blood. Ragelle et al. (87)constructed PEGylated chitosan-based NPs to deliver siRNAs and confirmed that these combined NPs achieved high gene silencing, low cytotoxicity, and high stability in B16 melanoma cells expressing luciferase.

Monolayer cell culture models *in vitro* do not predict the efficiency of *in vivo* clinical studies, and in fact melanoma cells establish interactions with their neighbors in a three-dimensional (3D) space. 3D culture models (melanoma spheroids) recreate tumor heterogeneity by reconstructing natural gradients such as hypoxic environment (88), however, there are only a few reports of PDT on melanoma spheroids mimic tumor architecture.

PDT is less effective against pigmented melanoma than against NMSC because the main function of melanin synthesized in melanocytes is to protect against UV-induced damage, which prevents light from efficiently reaching the targets. Naidoo et al. (89) reported that PSs including verteporfin, halogenated porphyrins, 5-ALA, ruthenium porphyrins, and Pcs have been applied for PDT in MM. Cai et al. (90) found that 5-ALA PDT could significantly inhibit the survival of MM-A375 and A431 cells in a concentration-dependent and time-dependent manner, and the mechanism of action was related to the downregulation of Bcl-2 and upregulation of Bax and cleaved-PARP.

**Figure 3:**
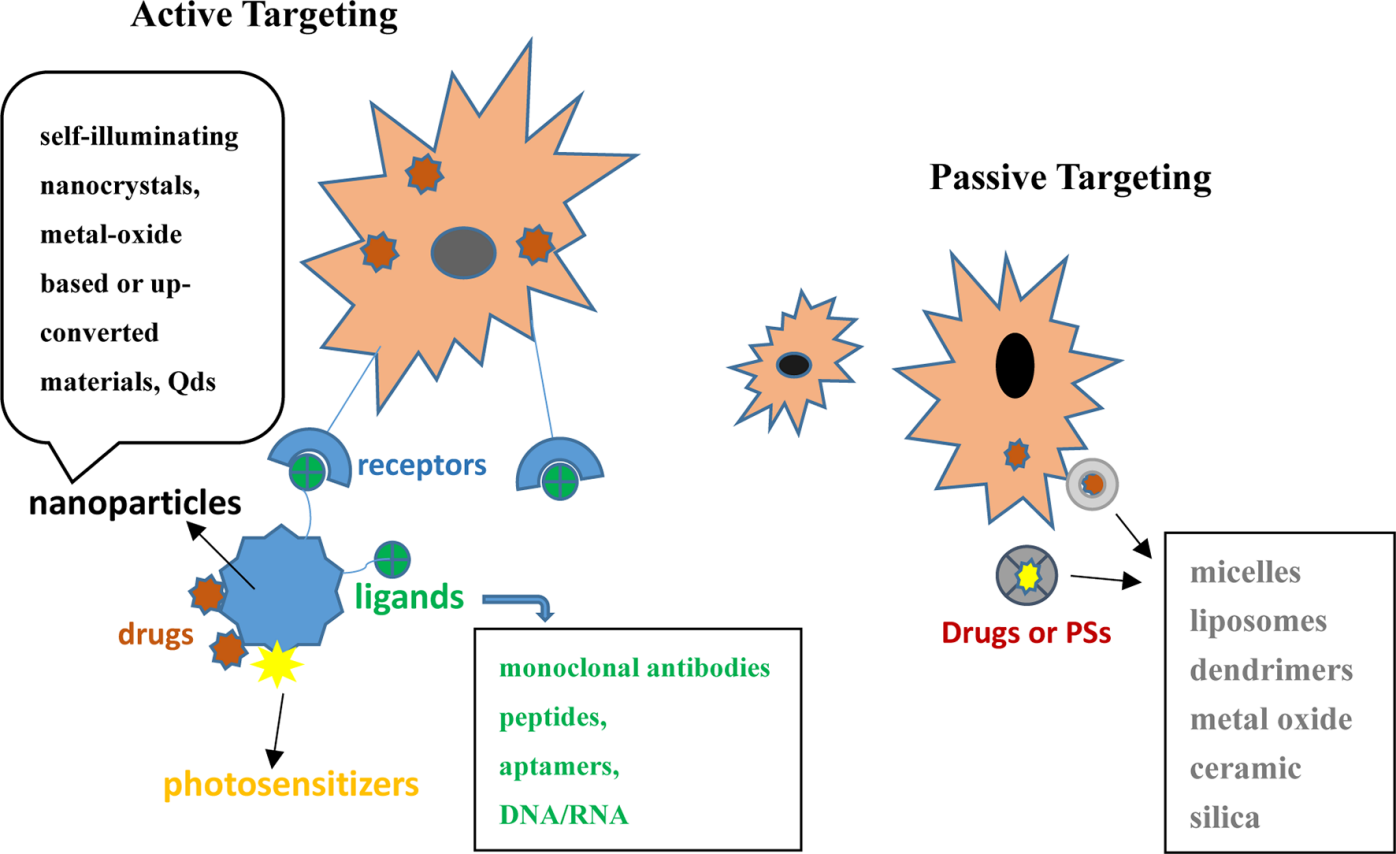
Active and passive forms of PSs or chemotherapeutics in combination with nanocarriers in PDT for skin cancer.

The incorporation of antibodies or targeting molecules to NPs improved passive or active PS delivery to target tumor cells. Functionalized NP platforms are beneficial for enhancing PS drug delivery, as presented in **Figure 3**.

Nanocarriers can be divided into two types based on whether they are absorbed passively or actively. Passive drug delivery systems included micelles, LPs, dendrimers, metal oxide, ceramic, and silica (91).

Although the pigmentation of melanoma reduces the efficacy of PDT, researchers (92) used phenylthiourea (a melanin synthesis inhibitor) to inhibit melanin synthesis and obtain de-pigmented melanoma cells, and these cells were then co-incubated with LPs encapsulating sodium ferrous chlorophyllin (Fe-CHL). Transmission electron microscopy revealed an increase in the cellular uptake, and the compounds mainly accumulated in mitochondria and nuclei, thus increasing the efficacy of Fe-CHL–mediated PDT. Lee et al. (93) demonstrated that chitosan-coated LPs improved the stability and permeation of ICG, increased the cellular uptake of agent, and enhanced the efficacy of topical PDT in B16-F10 melanoma cells. Polish scientists designed a co-delivery system combining IR-768 with daunorubicin *via* mPEG-b-PLGA micelles, and the dual drug-loaded delivery system enhanced ^1^O_2_ generation during PDT in A375 cells (94). Chen et al. (95) encapsulated palladium porphyrin (PdTCPP) in layered metal oxide NPs (LDH), which improved the solubility of hydrophobic PS-PdTCPP and increased the biocompatibility and stability of the nanocomposites. Because LDH had higher loading capability, the surface could be modified with different functional groups. PdTCPP-LDH provided excellent anti-cancer efficacy during PDT in B16F10 cells.

NPs can be functionalized with targeting molecules that can selectively bind to receptors overexpressed on tumor cells, leading to enhanced PS/drug uptake. Monoclonal antibodies, peptides, aptamers, DNA/RNA, and other targeting molecules have been used for active targeting in PDT. In this process, modalities such as Qds, self-illuminating nanocrystals, and metal oxide-based or upconverting material have been selected as nanocarriers (96).

The peak absorption of melanin pigment is around 335 nm, and absorption is almost completely attenuated at the wavelengths longer than 700 nm. GNPs irradiated with NIR, tunable optics and photothermal properties can exert synergistic effects with PSs in PDT. Researchers assessed the dual efficacy of PDT/PTT using complexes of ZnPc attached to AuNRs *in vitro* with B16F10 melanotic cells and B16G4F amelanotic cells under irradiation at 635 nm. The results indicated that the photodynamic activity and photothermal effect could eliminate more than 90% of melanoma cells (97). Campu et al. (98)designed a near-infrared irradiation-responsive dual PTT/PDT therapeutic nanosystem featuring gold nanobipyramids (AuBPs) loaded with ICG. When the nanosystem was simulated by NIR, the ^1^O_2_ yield was doubled and the PTT effect was strongly increased compared with the findings for AuBPs alone. Urszula et al. (99) designed xanthene-originated RB co-encapsulated with upconverting NaYF4 NPs co-doped with lanthanide ions (Er3+ [2%] and Yb3+ [20%]) and then engineered them with PLGA and non-ionic surfactants (Span 80 and Cremophor A25). The outcome indicated that the double-core nanoplatform for the co-delivery of hybrid fluorophores had excellent selectivity, biocompatibility, and PDT effects in human melanoma cells (Me-45 and MeWo).

## Summary

Thus far, numerous studies have investigated the combined efficacy of PSs with nanomaterials in PDT. Although the actual targeted transport efficiency of conventional PSs *in vivo* is hindered because of complex conjugated structures, poor water solubility and variational biological environment (e.g.pH, oxygen, nutrients), nano-PSs strategies open new ways for efficient PDT. Some local and superficial skin cancers and precancerosis are absolute indications for nanotech-mediated PDT. Furthermore, nanocarriers can better control drug release and significantly improve ROS-generating ability.

PDT still faces many challenges in clinical application. The excitation wavelength of PSs is mainly concentrated in UV or VIS, but biological tissues have strong absorption and scattering effect on light in the wavelength range, resulting in weak penetration ability, which fail to be directly applied in deep tumors or large solid tumors. NIR with preeminent penetration depth at 700-1000 nm, is an ideal PDT excitation light. Unfortunately, compared with UV-VIS, the molar absorption coefficient of PSs with the absorption peak in NIR is usually small, so one-photon excitation (OPE) PDT effect is non-ideal under NIR excitation with a safe radiation dose. Two-photon excitation (TPE) PDT has excellent spatial selectivity, and can achieve accurate target positioning, however, TPE-PDT PSs mainly occurs in the focal region, with small two-photon absorption cross section, thus resulting in low efficiency of TPE PDT, even using high-energy NIR technique, PDT efficacy cannot be improved significantly. Compared with TPE, the upconversion process does not need a high-power laser (98), and conventional continuous lasers can make UCNPs emit short-wavelength light, so as to reduce tissues damage caused by high-power light sources (100), but the photoquantum yield of UCNPs is relatively low. Weighing the pros and cons of various nanomaterials, it is crucial that constructing the ideal PSs compounds with the characteristics including high degree of chemical purity and stability, easy solubility in the tissues, high photochemical reactivity and photosensitive effect, and the maximum absorption of light should be at wavelengths 600 nm-800 nm, excellent selectivity and minimal cytotoxicity.

Due to oxygen consumption and microvascular damage, the anti-tumor efficacy of PDT is limited by tumorous hypoxic state. Scholars have designed a variety of nanodelivery systems to enhance the blood oxygen content of tumor sites, including direct oxygen delivery (by hemoglobin or perfluorocarbons NPs), catalytic oxygen production strategies (catalase oxygen-producing nanodelivery system), and so on. Direct oxygen delivery strategy and enzyme-catalyzed oxygen production strategy are affected by delivery efficiency and substrate concentration (101), in order to further improve the efficacy of oxygen-enhanced PDT, researchers used responsive materials to react with water *in vivo* to generate oxygen, which can effectively improve hypoxic internal environment. PDT has become one of the most important clinical therapies in dermatology. It is believed that with the development and integration of nanomedicine, biology and optics, more and more dermatological patients will benefit from PDT.

## Author Contributions

ZP and DL helped draft the manuscript. All authors contributed to the article and approved the submitted version.

## Funding

This work was supported by the National Natural Science Foundation of China (No. 81803157).

## Conflict of Interest

The authors declare that the research was conducted in the absence of any commercial or financial relationships that could be construed as a potential conflict of interest.

## Publisher’s Note

All claims expressed in this article are solely those of the authors and do not necessarily represent those of their affiliated organizations, or those of the publisher, the editors and the reviewers. Any product that may be evaluated in this article, or claim that may be made by its manufacturer, is not guaranteed or endorsed by the publisher.

## Abbreviations

NPs, nanoparticles; EPR, enhanced permeability and retention effect; LPs, liposomes; SPIONs, super-paramagnetic iron oxide NPs; Qds, quantum dots; PEG, polyethylene glycol; AuNRs, gold nanorods; AlPcS, aluminum Pcs; PL, photoluminescence; FRET, fluorescence resonance energy transfer; Ce6, chlorin e6; UCNPs, upconverting NPs; NIR, near-infrared light; PTT, photothermal therapy; Dox, doxorubicin; TPP, tetraphenylporphyrin; PhA, pheophorbide A; CAR, carotene; SCC, squamous cell carcinoma; cSCC, Cutaneous SCC; BCC, basal cell carcinoma; MM, metastatic melanoma; NMSC, non-melanoma skin cancer; PpIX, protoporphyrin IX; AFL, ablative fractional laser; MAL, aminolevulinate.
